# Herpes zoster prophylaxis: Essential for treating newly diagnosed multiple myeloma patients

**DOI:** 10.1002/cam4.5215

**Published:** 2022-09-20

**Authors:** Wen‐Ying Lin, Chun‐Kuang Tsai, Chiu‐Mei Yeh, Tin Chian, Yao‐Chung Liu, Hao‐Yuan Wang, Po‐Shen Ko, Ting‐An Lin, Liang‐Tsai Hsiao, Po‐Min Chen, Jyh‐Pyng Gau, Chia‐Jen Liu

**Affiliations:** ^1^ Department of Medicine Taipei Veterans General Hospital Taipei Taiwan; ^2^ Division of Hematology, Department of Medicine Taipei Veterans General Hospital Taipei Taiwan; ^3^ School of Medicine National Yang Ming Chiao Tung University Taipei Taiwan; ^4^ Institute of Public Health National Yang Ming Chiao Tung University Taipei Taiwan; ^5^ Institute of Emergency and Critical Care Medicine National Yang Ming Chiao Tung University Taipei Taiwan

**Keywords:** herpes zoster, multiple myeloma, prophylaxis

## Abstract

**Background:**

Multiple myeloma (MM) is known for its immune disturbance and patients suffering from MM are thus vulnerable to opportunistic infections, including herpes zoster (HZ). As HZ infection remarkably affects patients' quality of life and poses huge economic burdens on the health system, we aim to identify the risk factors of HZ infection and evaluate the effects of different dosages, types, and durations of anti‐HZ prophylaxis drugs to prevent HZ infection.

**Methods:**

551 MM patients at Taipei Veterans General Hospital in Taiwan between January 1, 2009 and August 31, 2021 were restrospectively analyzed. The patients' baseline characteristics were recorded. The primary endpoint of the study was the incidence of HZ infection among the studied patient population. Due to the lack of cost coverage from Taiwanese public health insurance on HZ prophylaxis drugs, the use of anti‐HZ drugs mainly depends on physicians' preferences and patients' choices.

**Results:**

In our study, prophylaxis was given to 283 of the patients. In the multivariate analysis, we included non‐prophylaxis, age ≥ 60, corrected serum calcium ≥12 mg/dl, serum creatinine ≥2 mg/dl, serum β2‐microglobulin ≥5500 mg/L, autologous stem cell transplant (SCT), and allogeneic SCT for analysis. Our results demonstrated that the non‐prophylaxis group (HR: 2.37, 95% CI 1.57–3.57) and patients receiving autologous SCT (HR: 2.22, 95% CI 1.28–3.86) and allogeneic SCT (HR: 5.12, 95% CI 1.13–23.22) had higher risk of HZ infection. The difference in dosage and types of anti‐HZ drugs showed similar protective effects. In patients who stopped anti‐HZ prophylaxis before active cancer‐related treatment, a higher risk of getting HZ infection compared to the corresponding group was also observed (adjusted HR 3.09, 95% CI 1.35–7.07, *p* = 0.008).

**Conclusions:**

We concluded that MM patients should receive HZ prophylaxis drugs while receiving active cancer‐related treatment. Patients receiving SCT are also at high risk of getting HZ infection, even under prophylaxis.

## Graphical abstract

Patients with multiple myeloma should receive herpes zoster prophylaxis drugs while receiving active cancer‐related treatment. Those who receive stem cell transplants, either autologous or allogeneic, are also at high risk of getting herpes zoster infection, even under prophylaxis.

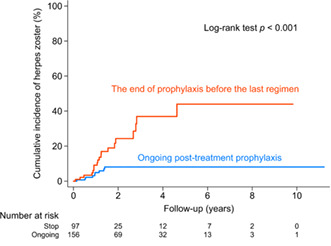

## 1 INTRODUCTION

Multiple myeloma (MM), known as the second most common hematological malignancy, accounts for about 1% of all cancer types worldwide.^1^, ^2^ It is characterized by suppressed production of normal immunoglobulins by the abnormal clone, which is thought to mainly disturb humoral immunity.^3^, ^4^ Since 1970, it has been shown to incapacitate cell‐mediated immunity^5^, ^6^ and have deregulation of adaptive T cell immunity.^7^ Pronounced imbalance of NK cell disorders was observed as well.^8^, ^9^, ^10^ These deficiencies together result in poor elimination of the virus and lead to immunodeficiency and consequent infections.^11^, ^12^ It is not surprisingly that patients with MM are recognized as immunocompromised and are vulnerable to opportunistic infections, including herpes zoster (HZ) and invasive fungal infection.^13^, ^14^, ^15^

Among episodes of infections, the sequala of HZ, such as postherpetic neuralgia (PHN), remains one of the major harassments affecting patients' quality of life. Nearly 10%–60% of patients with MM develops HZ infection after cancer‐related treatment,^16^, ^17^, ^18^, ^19^ especially the proteasome inhibitors,^20^, ^21^, ^22^ and monoclonal antibody, daratumumab,^23^ if remained without prophylactic treatment. This incidence has been reported to be around five times higher compared with the general population after being standardized by age and sex.^24^ PHN may persist for months to years after the resolution of an acute event,^25^ and can be profound and interfere with daily activities, leading to psychosocial impairment.^26^, ^27^ A recent cohort study demonstrated poor outcomes on health‐related quality of life (HRQL) and huge economic burdens after having HZ infection.^28^ As the median overall survival of MM patients has been prolonged to 10 years in those eligible for autologous stem‐cell transplantation,^29^ and in those transplant ineligible patients who receive induction therapy followed by adequate maintenance,^30^ prevention of HZ infection during treatment is thus considered essential.

Over the years, multiple studies have proposed that prophylactic use of antivirus drugs is beneficial for HZ prevention, with acyclovir dosage ranging from 200 mg once daily^31^, ^32^, ^33^ to 400 mg once daily,^31^, ^33^, ^34^ 400 mg twice daily,^35^ and 400 mg three times daily,^34^ or alternatively using valacyclovir^33^, ^36^ or famciclovir.^33^ Consistent with these studies, the National Comprehensive Cancer Network recommends that all MM patients treating with a proteasome inhibitor should receive HZ prophylaxis. However, there is currently no consensus on the dosage and duration of HZ prophylaxis. Furthermore, patients with treatments other than a proteasome inhibitor were less assessed for the immediate and sustained effects of HZ infection prophylaxis.

In this study, we aim to determine whether preventive use of acyclovir/valacyclovir in patients with MM produces meaningful beneficial clinical outcomes and whether different dosages and durations produce different effects. We conducted a retrospective cohort study to compare clinical outcomes in MM patients receiving prophylaxis or not during a 10‐year follow‐up after diagnosis.

## 2 METHODS

### 2.1 Study population

This is a retrospective cohort study. The study enrolled patients who were newly diagnosed with MM at Taipei Veterans General Hospital between January 2009 and August 2021. All patients were age 20 or older to be eligible for this study. Diagnosis of MM followed International Myeloma Working Group criteria,^37^ with documented bone marrow biopsies. Patients with smoldering myeloma, monoclonal gammopathy of undetermined significance (MGUS), solitary plasmacytomas and amyloidosis without meeting active IMWG‐defined myeloma were excluded from this study. The study was approved by the Taipei Veterans General Hospital institutional review board and the ethics committee (no. 2021‐03‐006CC).

### 2.2 Data collection

We retrospectively reviewed patients' medical records and assembled patients' characteristics compromised of age, sex, Eastern Cooperative Oncology Group (ECOG) performance status, disease classification based on Durie‐Salmon stage and the International Staging System (ISS), immunoglobulin heavy and light chain isotype, and whether the patient has had an organ transplant. Comorbidities including autoimmune disease, human immunodeficiency virus (HIV), heart failure, chronic pulmonary disease, and diabetes mellitus were also collected. The percentage of plasma cells in bone marrow was also recorded. Initial laboratory data, including hemoglobin level, platelet count, serum levels of albumin, corrected calcium, creatinine, lactate dehydrogenase (LDH), β2‐microglobulin, and free light chain ratio were all obtained at the time of diagnosis of MM. As for treatment, we gathered each patient's therapeutic regimens, including the proteasome inhibitor bortezomib, carfilzomib, ixazomib, and the anti‐CD38 agent daratumumab, as well as whether the patient received an autologous/allogeneic SCT.

### 2.3 HZ definition and prophylaxis treatment

Potential cases of HZ infection were identified by extracting records on all inpatient and outpatient encounters occurring between January 2009 and August 2021. We defined the HZ infection cases based on a description in the medical records. The starting date of vesicle development or HZ infection diagnosis date was documented. Clinical records were reviewed for prophylactic drug type, dosage, and duration.

### 2.4 Statistical analysis

We analyzed the baseline characteristics of MM patients using the total number (*n*) and the proportion (%). Categorical variables were compared in categorical forms by using chi‐square tests. The primary endpoint of the study was the incidence of HZ infection among the studied patient population. The accumulated HZ infection incidence from the date of MM diagnosis until the date of HZ infection diagnosis, death, loss of follow‐up, or the end of August 2021. The Kaplan–Meier curve (log rank test) was adopted to analyze the impact of the use of HZ prophylaxis on the cumulative incidence of HZ infection. It was used to measure the fraction of patients suffering from HZ infection for a certain amount of time after the initiation of the treatment. Differences between prophylaxis were tested using a log‐rank test. A Cox proportional hazards model was used to assess the risk factors for HZ infection. Hazard ratios (HRs) and 95% confidence intervals (CIs) for HZ infection risks were calculated, respectively, for age, sex, ECOG performance status, comorbidities, and initial laboratory data to assess the risk factor of HZ infection. As for prophylaxis and treatment, such as bortezomib, autologous SCT or allogeneic SCT, we used a time‐dependent covariate in the Cox proportional hazards model to prevent immortal time bias. A univariate model was used to identify risk factor. Those factors whose *p* value <0.1 were included in our multivariate analysis.

Furthermore, we applied a Cox proportional hazards model to measure the associations between HZ infection and mortality risks, controlling for potential confounding factors in the multivariate model. The episodes of HZ infection and prophylaxis period were estimated using a time‐dependent Cox proportional hazards models to prevent immortal time bias. The whole data management and statistical analysis were conducted using SAS 9.4 software (SAS Institute Inc.) and STATA statistical software, version 15.1 (StataCorp). The significance level was set as *p* value <0.05, based on a two‐tailed test, throughout the study.

## 3 RESULTS

### 3.1 Clinical characteristics of the study population

**FIGURE 1:**
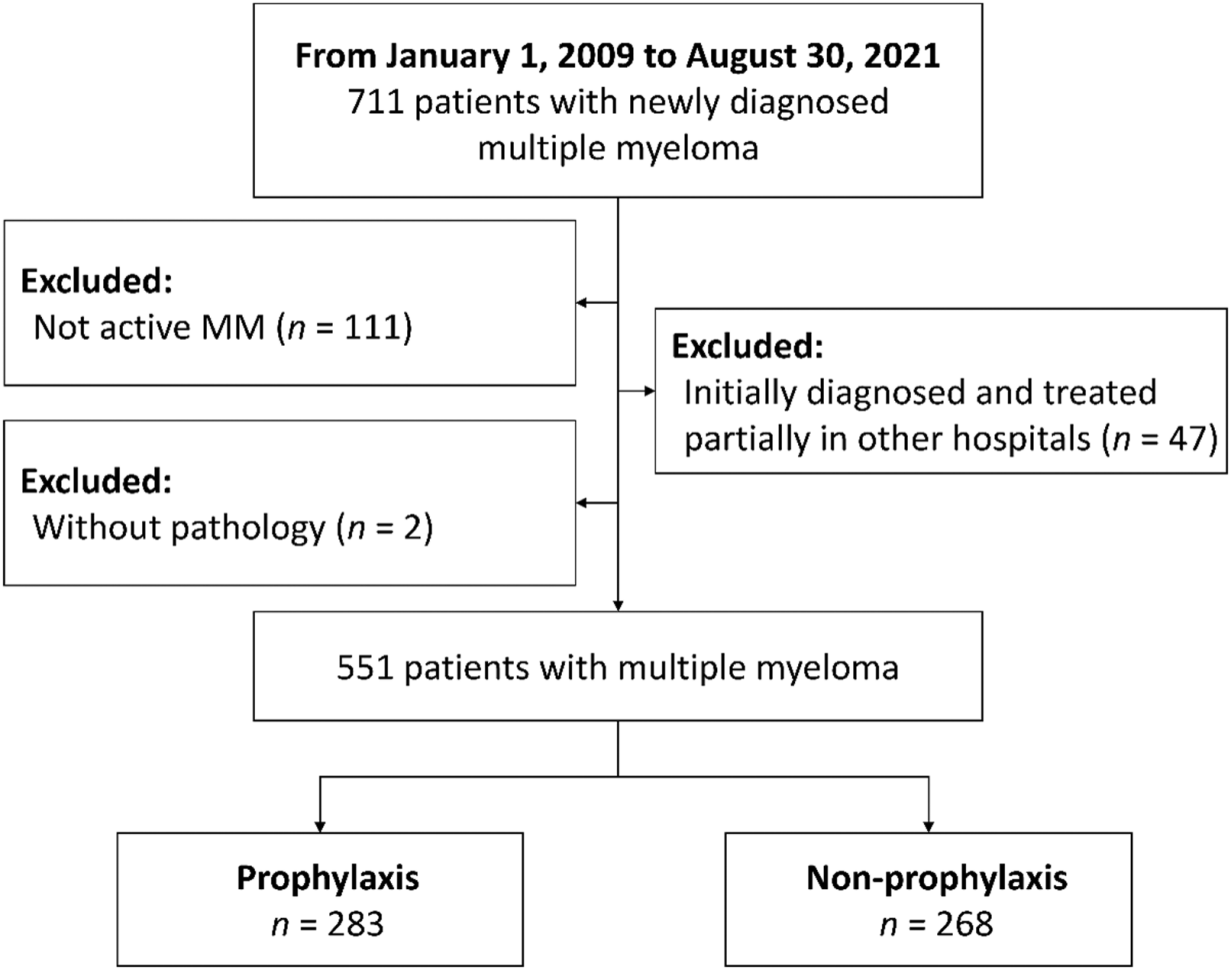
Patient selection flowchart. A total of 771 patients with newly diagnosed multiple myeloma were checked; 551 patients were included in this cohort study and were categorized into two groups according to the prophylaxis use of anti‐HZ drugs or not.

Our study enrolled 711 patients with newly diagnosed MM and measurement of prophylaxis treatment of HZ, from January 1, 2009 to August 30, 2021. Of them, 111 patients who were not active MM (solitary plasmacytomas, MGUS, smoldering myeloma, and amyloidosis), 47 patients who were initially diagnosed and treated partially in other hospitals, and two patients without pathology were excluded. The final study cohort included 551 MM patients. Among them, 283 (51.4%) patients used either acyclovir or valacyclovir to prevent HZ infection, while the remaining 268 (48.6%) patients did not receive prophylaxis treatment (Figure 1). The baseline characteristics of the study population are shown in Table 1. The median age of the cohort was 69 (range 23–96 years), 416 (75.5%) patients were age 60 or older, and 328 (59.5%) were male.

**TABLE 1:** Baseline patient characteristics of multiple myeloma

Characteristics	Total *n* = 551	Prophylaxis *n* = 283	Non‐prophylaxis *n* = 268	*p* value
Median age, years (range)	69 (23–96)	66 (23–95)	71 (31–96)	0.002
Age ≥ 60 years	416 (75.5%)	208 (73.5%)	208 (77.6%)	0.262
Sex (male)	328 (59.5%)	150 (53.0%)	178 (66.4%)	0.001
ECOG				
0–1	302 (54.8%)	174 (61.5%)	128 (47.8%)	< 0.001
≥2	227 (41.2%)	92 (32.5%)	135 (50.4%)	
Unknown	22 (4.0%)	17 (6.0%)	5 (1.9%)	
Durie‐Salmon stage				
I	192 (34.9%)	104 (36.8%)	88 (32.8%)	0.064
II	136 (24.7%)	58 (20.5%)	78 (29.1%)	
III	223 (40.5%)	121 (42.8%)	102 (38.1%)	
ISS stage				
I	115 (20.9%)	67 (23.7%)	48 (17.9%)	0.186
II	218 (39.6%)	112 (39.6%)	106 (39.6%)	
III	216 (39.2%)	103 (36.4%)	113 (42.2%)	
Unknown	2 (0.4%)	1 (0.4%)	1 (0.4%)	
Heavy chain				
IgA	148 (26.9%)	78 (27.6%)	70 (26.1%)	0.519
IgG	283 (51.4%)	139 (49.1%)	144 (53.7%)	
Others	120 (21.8)	66 (23.3)	54 (20.2)	
Light chain				
Kappa	291 (52.8%)	154 (54.4%)	137 (51.1%)	0.119
Lambda	250 (45.4%)	127 (44.9%)	123 (45.9%)	
Others	10 (1.8%)	2 (0.7%)	8 (3.0%)	
Organ transplant	2 (0.4%)	2 (0.7%)	0 (0.0%)	0.500
Comorbidities				
Diabetes mellitus	104 (18.9%)	45 (15.9%)	59 (22.0%)	0.067
Heart failure	90 (16.3%)	35 (12.4%)	55 (20.5%)	0.010
Pulmonary	67 (12.2%)	29 (10.3%)	38 (14.2%)	0.158
Autoimmune disease	17 (3.1%)	9 (3.2%)	8 (3.0%)	0.895
HIV	1 (0.2%)	0 (0.0%)	1 (0.4%)	0.486
Laboratory data				
Plasma cells of bone marrow ≥60%	268/498 (53.8%)	147/258 (57.0%)	121/240 (50.4%)	0.142
Hemoglobin <10 g/dl	326/550 (59.3%)	160/282 (56.7%)	166/268 (61.9%)	0.215
Platelet <100,000/μl	102/549 (18.6%)	47/281 (16.7%)	55/268 (20.5%)	0.253
Serum albumin <3.5 g/dl	310/541 (57.3%)	151/277 (54.5%)	159/264 (60.2%)	0.179
Corrected serum calcium ≥12 mg/dl	50/516 (9.7%)	31/263 (11.8%)	19/253 (7.5%)	0.101
Serum creatinine ≥2 mg/dl	146/546 (26.7%)	74/280 (26.4%)	72/266 (27.1%)	0.866
Lactate dehydrogenase ≥250 U/L	154/527 (29.2%)	77/273 (28.2%)	77/254 (30.3%)	0.595
Serum β2‐microglobulin ≥3500 mg/L	215/511 (42.1%)	154/265 (58.1%)	166/246 (67.5%)	0.029
Light chain ratio > 100	182/493 (36.9%)	115/275 (41.8%)	67/218 (30.7%)	0.011
Stem cell transplantation (SCT)				
Autologous SCT	113 (20.5%)	80 (28.3%)	33 (12.3%)	< 0.001
Allogeneic SCT	6 (1.1%)	1 (0.4%)	5 (1.9%)	0.114
Treatment				
Bortezomib	330 (59.9%)	235 (83.0%)	95 (35.5%)	< 0.001
Carfilzomib	15 (2.7%)	12 (4.2%)	3 (1.1%)	0.024
Ixazomib	7 (1.3%)	7 (2.5%)	0 (0.0%)	0.015
Daratumumab	35 (6.4%)	33 (11.7%)	2 (0.8%)	< 0.001

Abbreviations: ECOG, Eastern Cooperative Oncology Group performance; HIV, human immunodeficiency virus; ISS, International Staging System; SCT, stem cell transplant.

Most of the patients were categorized as ECOG 0–1 (54.8%). Durie‐Salmon stages I, II, III accounted for 34.9%, 24.7%, and 40.5%, whereas 20.9%, 39.6%, and 39.2% of the patients constituted ISS stages I, II, and III, respectively. A total of 283 (51.4%) patients had MM of the IgG type and 148 (26.9%) patients had IgA type. Regarding comorbidities, 104 (18.9%) patients had diabetes mellitus, and 90 (16.3%) had heart failure. Plasma cells in bone marrow equal to or more than 60% were found in 53.8%, and hemoglobin of less than 10 g/dl was documented as 59.3%. For treatment, 113 (20.5%) patients received autologous SCT, and six (1.1%) underwent allogeneic SCT. The use of proteasome inhibitors, including bortezomib (59.9%), carfilzomib (2.7%), and ixazomib (1.3%), and the anti‐CD38 daratumumab (6.4%) were also recorded.

### 3.2 Differences in clinical demographics

In groups between patients with anti‐HZ prophylaxis and without prophylaxis, we found that patients who received prophylaxis were younger, with a median age of 66, while non‐prophylaxis group had older patients, with a median age of 71 (*p* = 0.002). The prophylaxis group was also predominantly female (*p* = 0.001) and had a better ECOG (*p* < 0.001). Patients with heart failure seemed to have received fewer prophylaxis drugs (*p* = 0.010). A lower percentage of prophylaxis was observed in patients with serum β2‐microglobulin ≥3500 mg/L (*p* = 0.029), whereas a higher percentage was noted in patients with light chain ratio > 100 (*p* = 0.011). 80 out of 113 patients (*p* < 0.001) received prophylaxis in the autologous SCT group. Six patients received allogeneic SCT, but only one of them (*p* = 0.114) received prophylaxis anti‐HZ drugs. In patients treated with either proteasome inhibitors or anti‐CD38, higher ratios of using prophylaxis anti‐HZ drugs were also noted. 235 out of 330 patients were prescribed with prophylaxis drugs in the group using bortezomib (*p* < 0.001), as were 12 out of 15 (*p* = 0.024) in the carfilzomib group, 7 out of 7 (*p* = 0.015) in the ixazomib group, and 33 out of 35 (*p* < 0.001) in the daratumumab group (Table 1). In prophylaxis group, all the patients started HZ prophylaxis when they initiated their first line of cancer‐related treatment. In other words, patients were mostly analyzed during the phase of disease activation. Median (IQR) duration of anti‐HZ prophylaxis drugs was 8.6 (4.2–13.3) months.

### 3.3 Risk factors of herpes zoster infection

**FIGURE 2:**
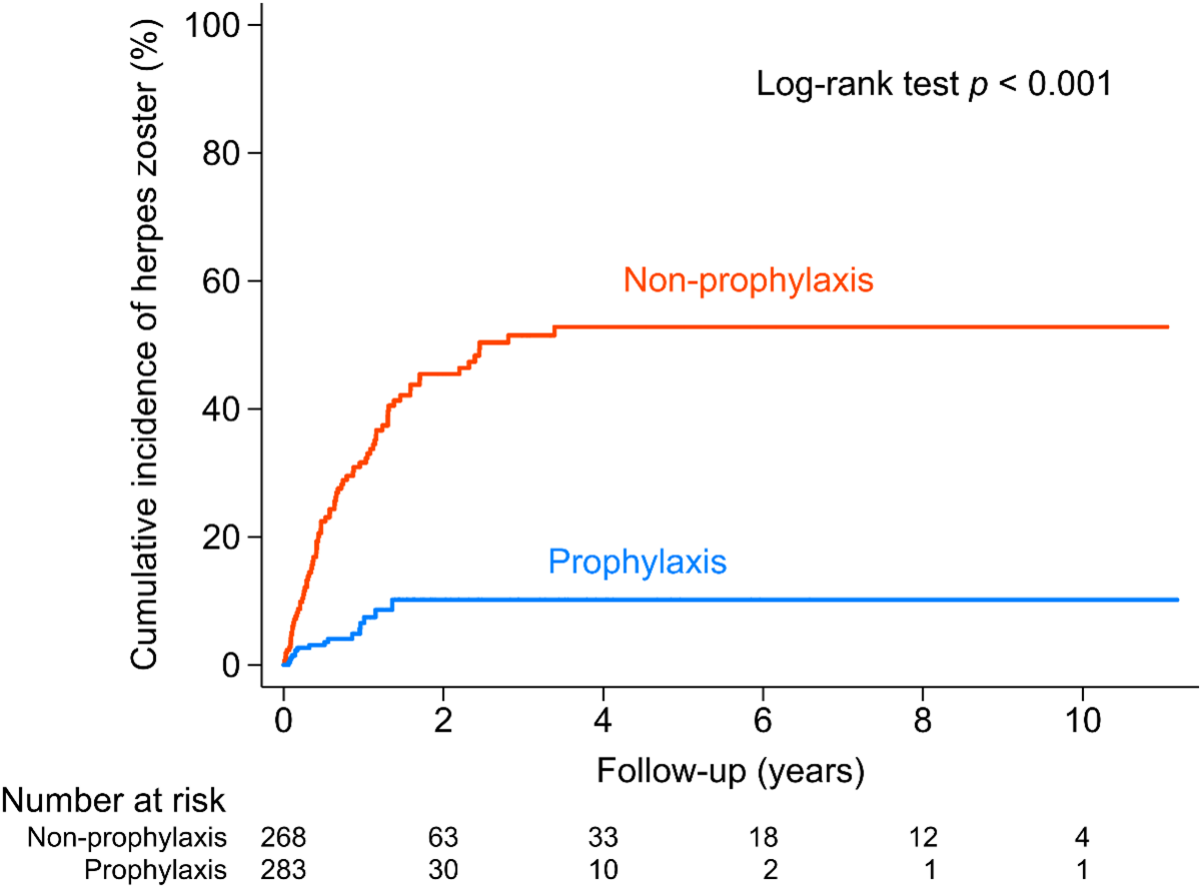
Cumulative incidence of herpes zoster in patients with multiple myeloma. Orange line represents the non‐prophylaxis group; blue line, prophylaxis group.

Risk factors of getting herpes zoster infection were analyzed (Table 2). In the univariate analysis, non‐prophylaxis (HR 2.29), serum creatinine ≥2 mg/dl (HR 1.57), serum β2‐microglobulin ≥5500 mg/L (HR 1.47) and receiving either autologous SCT (HR 2.04) or allogeneic SCT (HR 11.01) were associated with a higher rate of HZ infection (Table 2). In the multivariate analysis, non‐prophylaxis (adjusted HR 2.37, 95% CI 1.57–3.57, *p* < 0.001), receiving autologous SCT (adjusted HR 2.22, 95% CI 1.28–3.86, *p* = 0.005) and receiving allogeneic SCT (adjusted HR 5.12, 95% CI 1.13–23.22, *p* = 0.034) were associated with a higher rate of HZ infection. These results suggest that the use of prophylaxis drugs and a regimen containing transplantations or not are major factors for determining the risk of HZ infection. Because transplantations were the ultimate treatment goal in eligible patients, the use of a prophylaxis drug might be the only manipulation we could modify. The cumulative incidence of HZ infection was significantly higher in the non‐prophylaxis group, compared to its prophylaxis counterpart (log‐rank test *p* < 0.001, Figure 2). Most HZ infection happened within the first year in both groups, which is compatible with a previous reported peak in the incidence of viral infection.^38^ In our study, most of the patients with HZ infection presenting with skin lesions over one or two adjacent dermatomes and resolved after the use of anti‐virus drugs. No mortality was noted due to HZ infection.

**TABLE 2:** Risk factors for herpes zoster in multiple myeloma patients

Predictive variables	Univariate analysis		Multivariate analysis^a^	
	HR (95% CI)	*p* value	HR (95% CI)	*p* value
Non‐prophylaxis^b^	2.29 (1.56–3.35)	< 0.001	2.37 (1.57–3.57)	<0.001
Age ≥ 60 years	0.73 (0.50–1.06)	0.097	0.77 (0.49–1.21)	0.260
Sex (male)	1.06 (0.74–1.51)	0.767		
ECOG ≥2	1.29 (0.90–1.86)	0.171		
Comorbidities				
Diabetes mellitus	1.26 (0.81–1.95)	0.311		
Heart failure	0.82 (0.47–1.43)	0.479		
Pulmonary	0.69 (0.35–1.36)	0.280		
Autoimmune disease	1.54 (0.63–3.77)	0.345		
HIV	–	0.985		
Laboratory data				
Plasma cells of bone marrow ≥60%	1.19 (0.81–1.74)	0.381		
Hemoglobin <10 g/dl	0.99 (0.69–1.41)	0.946		
Platelet <100,000/μl	0.89 (0.52–1.54)	0.687		
Serum albumin <3.5 g/dl	1.09 (0.76–1.56)	0.658		
Corrected serum calcium ≥12 mg/dl	1.71 (0.99–2.94)	0.054	1.38 (0.76–2.51)	0.296
Serum creatinine ≥2 mg/dl	1.57 (1.06–2.31)	0.023	1.48 (0.89–2.46)	0.132
Lactate dehydrogenase ≥250 U/L	0.88 (0.57–1.38)	0.587		
Serum β2‐microglobulin ≥5500 mg/L	1.47 (1.02–2.13)	0.041	1.34 (0.83–2.14)	0.228
ALC ≥1460	0.93 (0.65–1.32)	0.671		
Light chain ratio > 100	1.22 (0.84–1.79)	0.297		
Stem cell transplantation (SCT)				
Autologous SCT^b^	2.04 (1.29–3.24)	0.003	2.22 (1.28–3.86)	0.005
Allogeneic SCT^b^	11.01 (2.57–47.22)	0.001	5.12 (1.13–23.22)	0.034
Treatment				
Bortezomib^b^	0.89 (0.62–1.29)	0.545		
Carfilzomib^b^	3.33 (0.45–24.57)	0.239		
Ixazomib^b^	–			
Daratumumab^b^	0.99 (0.14–7.17)	0.993		

Abbreviations: CI, confidence interval; ECOG, Eastern Cooperative Oncology Group performance; HR, hazard ratio; SCT, stem cell transplant.

^a^ All factors with *p* < 0.1 in the univariate analysis were included in the Cox multivariate analysis.

^b^ Treatment was analyzed as a time‐dependent covariate in the Cox regression model.

### 3.4 Prophylaxis medication dosage

In the dosage analysis of prophylaxis medication, 49.8% of prophylaxis MM patients had received valacyclovir 500 mg per day, 35.6% received acyclovir 200 mg per day, while the remaining 14.6% received 400 mg acyclovir per day (Table 3). Two of them received lower dosage of anti‐HZ drugs due to poor renal function and thus were not listed in the table. The incidence rate of HZ infection was 6.5 cases per 100 person‐years of follow‐up in the 500 mg valacyclovir group, and 6.2 and 8.1 cases per 100 person‐years in the 200 mg and the 400 mg acyclovir groups, respectively. There was no significant difference in getting HZ infections between the different dosages of anti‐HZ drugs. HRs were 0.81 (95% CI 0.39–1.67, *p* = 0.569) and 1.33 (95% CI 0.59–2.99, *p* = 0.487) for the 200 mg and 400 mg acyclovir groups, respectively. As for comparison, patients without prophylaxis had incidence rate up to 20.6 cases per 100 person‐years (HR 3.28, 95% CI 2.05–5.26, *p* < 0.001).

**TABLE 3:** Incidence of herpes zoster in multiple myeloma patients with prophylaxis medicine

Prophylaxis medicine	*n*	Herpes zoster no.	Per 100 person‐years	Crude HR (95% CI)	*p* value
Valacyclovir 500 mg	140	22	6.5	Reference	
Acyclovir^a^					
200 mg	100	11	6.2	0.81 (0.39–1.67)	0.569
400 mg	41	8	8.1	1.33 (0.59–2.99)	0.487
Non‐prophylaxis	268	81	20.6	3.28 (2.05–5.26)	<0.001

Abbreviations: CI, confidence interval; HR, hazard ratio.

^a^ One patient received acyclovir 100 mg and one patient received acyclovir 600 mg.

### 3.5 Prophylaxis drug duration

**FIGURE 3:**
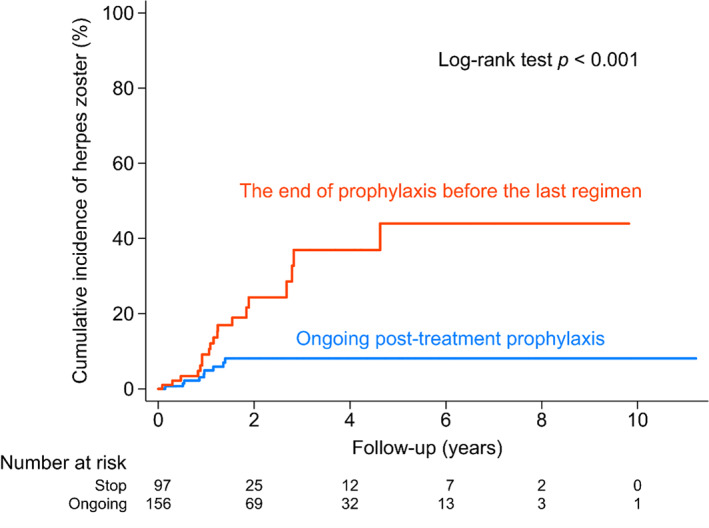
Cumulative incidence of herpes zoster in multiple myeloma patients receiving regimen with prophylaxis. Orange line represents patients ending anti‐HZ drugs prophylaxis before last cancer‐related regimen; blue line, patients continuing anti‐HZ drugs prophylaxis after end of active cancer‐related treatment.

As increased susceptibility to HZ infection has been linked to proteasome inhibitors, especially bortezomib,^20^, ^39^ we selected patients receiving first‐line regimens containing bortezomib for further analysis. We categorized them into two subgroups to study the effective duration of the prophylaxis drug. One group stopped taking the prophylaxis before first‐line cancer‐related treatment ended, while the other continued using the prophylaxis even after first‐line treatment. The cumulative incidence of HZ infection was remarkably higher in the group who ended prophylaxis before first‐line treatment (log‐rank test *p* < 0.001, Figure 3). This suggests that the use of prophylaxis should be continued at least until the end of cancer related treatment. However, a bimodal peak was observed in the incidence of viral infection.^38^ We did not observe the second peak. Additional analysis was done in an effort to identify how long the prophylaxis drug should be maintained after the end of cancer‐related treatment. Patients who had HZ infection had a median duration of 5.1 months (IQR 2.9–8.5) of using prophylaxis drugs after the end of their treatment regimen, while the other group, who did not get HZ infection, had a longer duration, with a median of 3.4 months (IQR 0.6–9.2) of using anti‐virus drugs (Figure S1). No significant difference between the two groups was observed (*p* = 0.172), which might be because the numbers of those who got HZ infevtion was small, and is not significant enough to reach a conslusion. Further studies regarding the duration of prophylaxis following cancer‐related treatment should be explored.

### 3.6 Risk factors of HZ infection during prophylaxis treatment

It has been reported that patients still get HZ infection even while undergoing prophylaxis treatment.^36^ We observed a similar phenomenon and therefore further analyzed the risk factors in patients who became infected with HZ while undergoing prophylaxis treatment (Table 4). In the univariate analysis, the end of prophylaxis before the last regiment (HR 4.27), corrected serum calcium ≥12 mg/dl (HR 2.94), serum creatinine ≥2 mg/dl (2.72), serum β2‐microglobulin ≥5500 mg/L (3.36) were related to a higher risk of getting HZ infection even during prophylaxis treatment. Interestingly, age ≥ 60 (HR 0.5) seemed to be a protective risk factor. In the multivariate analysis, the end of prophylaxis before the last regiment (HR 3.09) was the only risk factor.

**TABLE 4:** Risk factors for herpes zoster in multiple myeloma patients receiving regimen with prophylaxis

Predictive variables	Univariate analysis		Multivariate analysis^a^	
	HR (95% CI)	*p* value	HR (95% CI)	*p* value
The end of prophylaxis before the last regimen	4.27 (2.02–9.02)	<0.001	3.09 (1.35–7.07)	0.008
Age ≥ 60 years	0.50 (0.25–0.99)	0.048	0.26 (0.11–0.64)	0.003
Sex (male)	0.53 (0.26–1.10)	0.087	0.34 (0.14–0.84)	0.019
ECOG ≥2	1.88 (0.94–3.75)	0.074	0.99 (0.42–2.38)	0.988
Comorbidities				
Diabetes mellitus	0.59 (0.18–1.94)	0.385		
Heart failure	0.97 (0.30–3.17)	0.957		
Pulmonary	0.80 (0.19–3.33)	0.755		
Autoimmune disease	1.73 (0.41–7.22)	0.455		
HIV	–			
Laboratory data				
Plasma cells of bone marrow ≥60%	2.11 (0.94–4.75)	0.071	2.04 (0.71–5.84)	0.183
Hemoglobin <10 g/dl	1.27 (0.64–2.54)	0.497		
Platelet <100,000/μl	0.64 (0.19–2.09)	0.455		
Serum albumin <3.5 g/dl	1.99 (0.97–4.11)	0.062	0.86 (0.35–2.08)	0.731
Corrected serum calcium ≥12 mg/dl	2.94 (1.33–6.54)	0.008	2.03 (0.82–5.05)	0.128
Serum creatinine ≥2 mg/dl	2.72 (1.35–5.48)	0.005	2.39 (0.84–6.78)	0.103
Lactate dehydrogenase ≥250 U/L	1.51 (0.71–3.20)	0.280		
Serum β2‐microglobulin ≥5500 mg/L	3.36 (1.67–6.78)	0.001	2.62 (0.92–7.51)	0.072
Light chain ratio > 100	0.98 (0.49–1.95)	0.953		
Treatment				
Bortezomib^b^	1.79 (0.43–7.48)	0.427		
Carfilzomib^b^	–			
Ixazomib^b^	–			
Daratumumab^b^	1.86 (0.25–13.93)	0.548		

Abbreviations: CI, confidence interval; ECOG, Eastern Cooperative Oncology Group performance; HR, hazard ratio; SCT, stem cell transplant.

^a^ All factors with *p* < 0.1 in the univariate analysis were included in the Cox multivariate analysis.

^b^ Treatment was analyzed as a time‐dependent covariate in the Cox regression model.

### 3.7 Overall survival of MM patients and risk factor for all‐cause mortality

We further investigated the impact of HZ infection on MM patients' overall survival (Table 5). In the univariate analysis, having HZ infection (HR 1.43), taking non‐prophylaxis drugs (HR 1.38), being age ≥ 60 (HR 2.07), male (HR 1.71), ECOG ≥2 (HR 2.50), having diabetes mellitus (HR 1.54), heart failure (HR 2.04), pulmonary disease (HR 1.84) and HIV infection (HR 0.076) had a higher risk of death. In the laboratory data, hemoglobin <10 g/dl (HR 1.63), platelet <100,000/μl (HR 2.75), serum albumin <3.5 g/dl (HR 2.00), corrected serum calcium ≥12 mg/dl (HR 1.56), serum creatinine ≥2 mg/dl (HR 1.74), LDH ≥250 U/L (HR 1.96), and serum β2‐microglobulin ≥5500 mg/L (HR 2.13) at diagnosis were also related to higher mortality. Patients who received allogeneic stem cell transplantations (HR 5.03) and treatment including carfilzomib (HR 3.12) revealed a higher mortality rate as well. Interestingly, patients receiving autologous SCT had lower mortality rate (HR 0.62).

**TABLE 5:** Risk factors for mortality in multiple myeloma patients

Predictive variables	Univariate analysis		Multivariate analysis^a^	
	HR (95% CI)	*p* value	HR (95% CI)	*p* value
Herpes zoster infection^b^	1.43 (1.06–1.94)	0.020	1.25 (0.85–1.82)	0.257
Non‐prophylaxis^b^	1.38 (1.07–1.79)	0.013	1.28 (0.92–1.79)	0.147
Age ≥ 60 years	2.07 (1.52–2.83)	<0.001	1.70 (1.16–2.51)	0.007
Sex (male)	1.71 (1.32–2.21)	<0.001	1.66 (1.23–2.25)	0.001
ECOG ≥2	2.50 (1.95–3.20)	<0.001	1.99 (1.48–2.68)	<0.0001
Comorbidities				
Diabetes mellitus	1.54 (1.16–2.04)	0.003	1.18 (0.85–1.64)	0.324
Heart failure	2.04 (1.52–2.73)	<0.001	1.07 (0.74–1.55)	0.731
Pulmonary	1.84 (1.32–2.56)	<0.001	1.46 (0.97–2.20)	0.070
Autoimmune disease	1.14 (0.59–2.22)	0.694		
HIV	5.96 (0.83–42.70)	0.076	18.47 (2.38–143.47)	0.005
Laboratory data				
Plasma cells of bone marrow ≥60%	1.12 (0.86–1.45)	0.413		
Hemoglobin <10 g/dl	1.63 (1.26–2.12)	<0.001	1.01 (0.71–1.44)	0.962
Platelet <100,000/μl	2.75 (2.09–3.63)	<0.001	1.65 (1.15–2.36)	0.007
Serum albumin <3.5 g/dl	2.00 (1.53–2.60)	<0.001	1.67 (1.22–2.28)	0.001
Corrected serum calcium ≥12 mg/dl	1.56 (1.08–2.25)	0.017	1.29 (0.83–2.00)	0.261
Serum creatinine ≥2 mg/dl	1.74 (1.34–2.24)	<0.001	1.15 (0.80–1.66)	0.443
Lactate dehydrogenase ≥250 U/L	1.96 (1.51–2.54)	<0.001	1.50 (1.09–2.07)	0.013
Serum β2‐microglobulin ≥5500 mg/L	2.13 (1.65–2.74)	<0.001	1.39 (0.97–2.01)	0.077
Light chain ratio > 100	1.11 (0.85–1.46)	0.441		
Stem cell transplantation (SCT)				
Autologous SCT^b^	0.62 (0.44–0.87)	0.005	0.77 (0.50–1.19)	0.241
Allogeneic SCT^b^	5.03 (2.61–9.67)	<0.001	12.88 (6.10–27.21)	<0.001
Treatment				
Bortezomib^b^	0.80 (0.63–1.03)	0.084	1.08 (0.78–1.50)	0.625
Carfilzomib^b^	3.12 (1.78–5.49)	<0.001	0.35 (0.17–0.71)	0.004
Ixazomib^b^	1.57 (0.62–3.99)	0.344		
Daratumumab^b^	1.92 (0.97–3.81)	0.061	0.81 (0.35–1.86)	0.616

Abbreviations: CI, confidence interval; ECOG, Eastern Cooperative Oncology Group performance; HR, hazard ratio; SCT, stem cell transplant.

^a^ All factors with *p* < 0.1 in the univariate analysis were included in the Cox multivariate analysis.

^b^ Treatment was analyzed as a time‐dependent covariate in the Cox regression model.

In the multivariate analysis, HZ infection did not increase mortality rate, with adjusted HR 1.25 (95% CI 0.85–1.82, *p* = 0.257) after controlling for confounding factors. Patients in non‐prophylaxis group also showed no significantly predictive factor of mortality, with HR 1.28 (95% CI 0.92–1.79, *p* = 0.147). Patients age ≥ 60 (HR 1.70, CI 1.16–2.51, *p* = 0.007), male (HR 1.66, CI 1.23–2.25, *p* = 0.001), ECOG ≥2 (HR 1.99, CI 1.48–2.68, *p* < 0.001), HIV infection (HR 18.47, CI 2.38–143.47, *p* = 0.005), platelet <100,000/μl (HR 1.65, CI 1.15–2.36, *p* = 0.007), serum albumin <3.5 g/dl (HR 1.67, CI 1.22–2.28, *p* = 0.001), LDH ≥250 U/L (HR 1.50, CI 1.09–2.07, *p* = 0.013), and patients who received allogeneic stem cell transplantations (HR 13.81, CI 7.07–26.98, *p* < 0.001) had a higher mortality rate in the multivariate analysis. Interestingly, patients receiving autologous SCT has lower mortality (HR 0.62, CI 0.44–0.87, *p* = 0.005) in univariate analysis but this difference was not observed in multivariate analysis (HR 0.77, CI 0.50–1.19, *p* = 0.241). Carfilzomib was shown intriguingly to include a lower mortality rate (HR 0.35, CI 0.17–0.71, *p* = 0.004).

## 4 DISCUSSION

This retrospective study involving 551 patients with newly diagnosed MM, with a median follow‐up of 6 years, to our knowledge is the largest cohort study of this type in Asia. Our results demonstrate that there is no significant difference between valacyclovir and acyclovir in preventing HZ infection. Prophylaxis use of anti‐HZ drugs markedly lowers the risk of HZ infection and improves patient outcome.

Several retrospective studies have shown a positive relationship between prophylactic use of anti‐HZ drugs and a lower prevalence of HZ infection, especially in the group treated with bortezomib. This association was observed despite the dosage of prophylactic anti‐HZ drugs. For example, the average dose of acyclovir administrated ranged from 200 mg daily^31^, ^32^, ^33^ to 400 mg daily,^31^, ^33^, ^34^ 800 mg daily,^35^ or even to 1200 mg daily.^34^ Alternative use of valacyclovir^33^, ^36^ or famciclovir^33^ was also documented and turned out to have similar preventive action. A key limitation of previous studies was the small sample sizes, including around only 100 patients or even fewer, and were thus prone to measurement errors. In Leng's group,^36^ although larger patient numbers were obtained, participants existing chronic diseases, such as diabetes mellitus, were not documented, which might be a methodologic issue that resulted in the potential for reverse‐causation bias. To overcome these limitations, we followed patients with newly diagnosed MM in our hospital up to nearly 13 years and collected a total of 771 patients. Detailed comorbidities were also recorded. We further narrowed down our anti‐HZ drug choices to two easily accessible drugs: acyclovir, 200 mg daily, and 400 mg daily, or valacyclovir, 500 mg daily. No significant differences of getting HZ infection were noted between the groups of different doses or drugs. This finding might broaden our selection of anti‐HZ drugs. By documenting comorbidities of patients with MM, our study minimizes the immunocompromised effects of other cofounding diseases such as diabetes mellites. The use of individual‐level data for covariate adjustments renders our findings robust.

National Comprehensive Cancer Network guidelines suggest that all patients with MM treated with a proteasome inhibitor should receive HZ prophylaxis due to increased susceptibility to HZ.^20^, ^39^ However, how long prophylaxis should be administered was not well established. Although it is suggested to use prophylactic for at least 6 months to 1 year after autologous transplantation in other hematological malignancies,^40^, ^41^ and more than half of the VZV reactivation occurred within 1 year of proteasome inhibitor initiation,^42^ there is currently no consensus on how long patients should receive prophylactic medication when undergoing regimen other than transplant in patients with multiple myeloma. A recent study showed that HZ prophylaxis with low‐dose acyclovir over 12 months after ASCT is effective and well tolerated.^43^ In our study, we demonstrate that prophylaxis should be administered until at least the end of one's treatment regimen. Although further subgroup analysis did not show a significant different median duration between the two groups, we observed more scattered points beyond 1 year in the group without HZ infection. In previous studies, it has been shown that varicella zoster virus reactivations occurred late during treatment, with a median time of 6 months and a range 0–44 months, so physicians should be aware that HZ infection might occur 1 or 2 years later, even after an active cancer‐related treatment is discontinued. In addition, in patients receiving regimen with prophylaxis, we observed that older patients seemed to have lower risk of getting HZ infection. This might be due to higher mortality in older patients and thus their life might be terminated before we could observe HZ infection. More research is needed to answer these questions.

Unsurprisingly, an increased risk of HZ infection was noted in both post‐autologous and post‐allogeneic stem cell transplants in our study, compatible with the previous reported prevalence of 20%–53% in SCT recipients,^44^, ^45^, ^46^ whose T‐cell immunity was diminished.^47^ Nonetheless, in the group receiving autologous SCT, a little more than half of the patients (80/113) had anti‐HZ prophylaxis, and although they had an increased risk of HZ infection, it did not escalate mortality rate. On the other hand, in patients receiving allogeneic SCT in our study, only one of them received anti‐HZ prophylaxis. This is because concomitant use of acyclovir was documented to increase the risk of veno‐occlusive disease of the liver after hemopoietic cell transplantation.^48^, ^49^, ^50^ Also, under the concern of bone marrow suppression, VZV prophylaxis was not routinely used after hemopoietic cell transplantation in our hospital. However, an increased HZ infection risk and a higher mortality rate in were observed. This observation may suggest the beneficial giving HZ prophylaxis to patients undergoing SCT, but further study into the relationship is needed.

Another interesting issue is that we noted that patients with serum creatinine ≥2 mg/dl seemed to have increased HZ infection risk in univariate analysis, but not in multivariate analysis. Given within‐participant variation in stages of chronic kidney disease, which might alter the metabolism of anti‐HZ drugs, the true serum anti‐HZ drugs concentration was not validated. Although dosage recommendations for HZ treatment in patients with renal impairment from manufacturer's labeling was established based on previous studies,^51^, ^52^ no clear dosage adjustment for HZ prophylaxis in patients with renal impairment has been ratified. Further pharmacokinetics studies should be pursued for this subgroup of patients. Our result might also seem counterintuitive as patients with CKD should have lower clearance rate of anti‐HZ drugs, and thus have lower risk of getting HZ infection. However, it has been documented that CKD itself also contributed to the increasing prevalence of HZ infection,^53^, ^54^, ^55^ and the incidence of HZ even positively correlated with the progression in the stages of CKD.^55^ With the two factors, i.e. CKD stages and metabolism of anti‐HZ drugs, balancing out each other, we did not observe serum creatinine ≥2 mg/dl to be a risk factor in our multivariate analysis, and more detailed study is required.

In Table 2, it is also alluring to find that corrected serum calcium ≥12 mg/dl has higher HZ infection risk in univariate analysis but not in multivariate analysis. Corrected serum calcium ≥12 mg/dl was one of the criteria included in Durie‐almon staging system, which reflects disease severity. It has also been observed that hypercalcemia happened more frequently in patients with plasma cell leukemia, a late stage complication of myeloma,^56^, ^57^ and in patients with Durie‐Salmon stage III disease, circulating monomeric human calcitonin levels and corrected serum calcium were significantly higher than stage I.^58^ As for LDH, which has been shown as an important prognostic indicator of disease severity,^59^ might be affected by multiple factors such as hemolysis, and thus no significant difference was observed in our study. It is actually hard to define the association between disease severity i.e. stages and the degree of suppression of immunity system, as tumor and human immunity system has complicated crosstalk.^60^ In our study, we did not find direct relations between disease severity and HZ infection rate. Advanced studies regarding links between HZ infection and these biomarkers for disease severity might be worth pursued.

Our study has several limitations. First, it is a single medical center cohort, but we were able to reduce statistical errors using larger patient numbers. Second, this observational study does not allow us to rule out potential residual confounding, such as drug–drug interaction and drug compliance. Third, our study was based on study populations that comprised mainly generally Asians, so the generalizability of our results to other ethnic groups and to other patients with specific conditions is not possible. Forth, as the cost of VZV prophylaxis was currently not covered by public health insurance in Taiwan, the use of VZV prophylaxis will be mainly based on physicians' choice and patients' preference. In older patients, due to the concern of drug–drug interaction, poor compliance, and higher cormobidity due to side effects, such as neutorpenia related infection, VZV prophylaxis was not given. Also, if the patient was diagnosed in a late stage, complications such as death might occurred first before the use of prophylaxis drugs. These factors might also affect results. Fifth, our study contained only 6.4% of patients ever received daratumumab, a drug that has been reported to increase the risk of VZV reactivation.^23^ It is because daratumumab was not approved by Taiwanese Food and Drug Administration until late 2017. Thus, daratumumab was only used since then, which accounted for a small portion of populations in our study. Furthermore, as HZ vaccines have recently been well developed, with both high efficacy and safety for patients with MM,^61^, ^62^ and in patients receiving SCT,^63^, ^64^, ^65^ our study did not include the data about HZ vaccine in HZ prophylaxis due to the lack of availability of HZ vaccine in Taiwan. Further investigations are warranted.

Overall, our study shows a significant reduction in HZ infections when using prophylaxis anti‐HZ drugs in patients with MM undergoing treatment. The dosage and types of anti‐HZ drugs could generate similar protective action and can be chosen based on physicians' or patients' preference, and it should be continued at least to the completion of the cancer‐related regimen.

## AUTHOR CONTRIBUTIONS

**WenYing Lin:** Conceptualization (equal); data curation (equal); investigation (equal); methodology (equal); project administration (equal); writing – original draft (equal); writing – review and editing (equal). **Chun‐Kuang Tsai:** Conceptualization (equal); data curation (equal); methodology (equal); project administration (equal); writing – original draft (equal). **Chiu‐Mei Yeh:** Formal analysis (equal); investigation (equal); methodology (equal); writing – original draft (equal); writing – review and editing (equal). **Tin Chian:** Data curation (equal). **Yao‐Chung Liu:** Supervision (equal). **Hao‐Yuan Wang:** Supervision (equal). **Po‐Shen Ko:** Supervision (equal). **Ting‐An Lin:** Supervision (equal). **Liang‐Tsai Hsiao:** Supervision (equal). **Po‐Min Chen:** Supervision (equal). **Jyh‐Pyng Gau:** Supervision (equal). **Chia‐Jen Liu:** Conceptualization (equal); data curation (equal); formal analysis (equal); funding acquisition (equal); investigation (equal); methodology (equal); project administration (equal); resources (equal); software (equal); supervision (equal); validation (equal).

## FUNDING INFORMATION

This study was supported by grants from Taipei Veterans General Hospital (V110C‐195, V111C‐030 and V111B‐030), the Ministry of Science and Technology (MOST 104‐2314‐B‐075‐085‐MY2, MOST 109‐2314‐B‐075‐079‐MY2 and MOST 109‐2314‐B‐A49A‐503‐MY2), the Taiwan Clinical Oncology Research Foundation, the Szu‐Yuan Research Foundation of Internal Medicine, the Yen Tjing Ling Medical Foundation, the Melissa Lee Cancer Foundation, and the Chong Hin Loon Memorial Cancer and Biotherapy Research Center at National Yang‐Ming University. The funding sources had no role in the study design or conduct, or in the decision to submit it for publication.

## CONFLICTS OF INTEREST

The authors declare that they have no conflict of interest.

## CODE AVAILABILITY (SOFTWARE APPLICATION OR CUSTOM CODE)

Data management and all statistical analysis were performed using SAS 9.4 software (SAS Institute Inc., Cary, NC) and STATA statistical software, version 15.1 (StataCorp, College Station, TX).

## ETHICS STATEMENT

This study has been approved by the Institutional Review Board at Taipei Veterans General Hospital.

## Supporting information


Figure S1

## Data Availability

The retrospective data could be provided, if requested.
