# Supplementary figures and images for: Herpes zoster prophylaxis: Essential for treating newly diagnosed multiple myeloma patients
Source: Cancer Med. 2022 Sep 20;12(3):3013–26. doi: 10.1002/cam4.5215 (PMC9939124; doi:10.1002/cam4.5215)

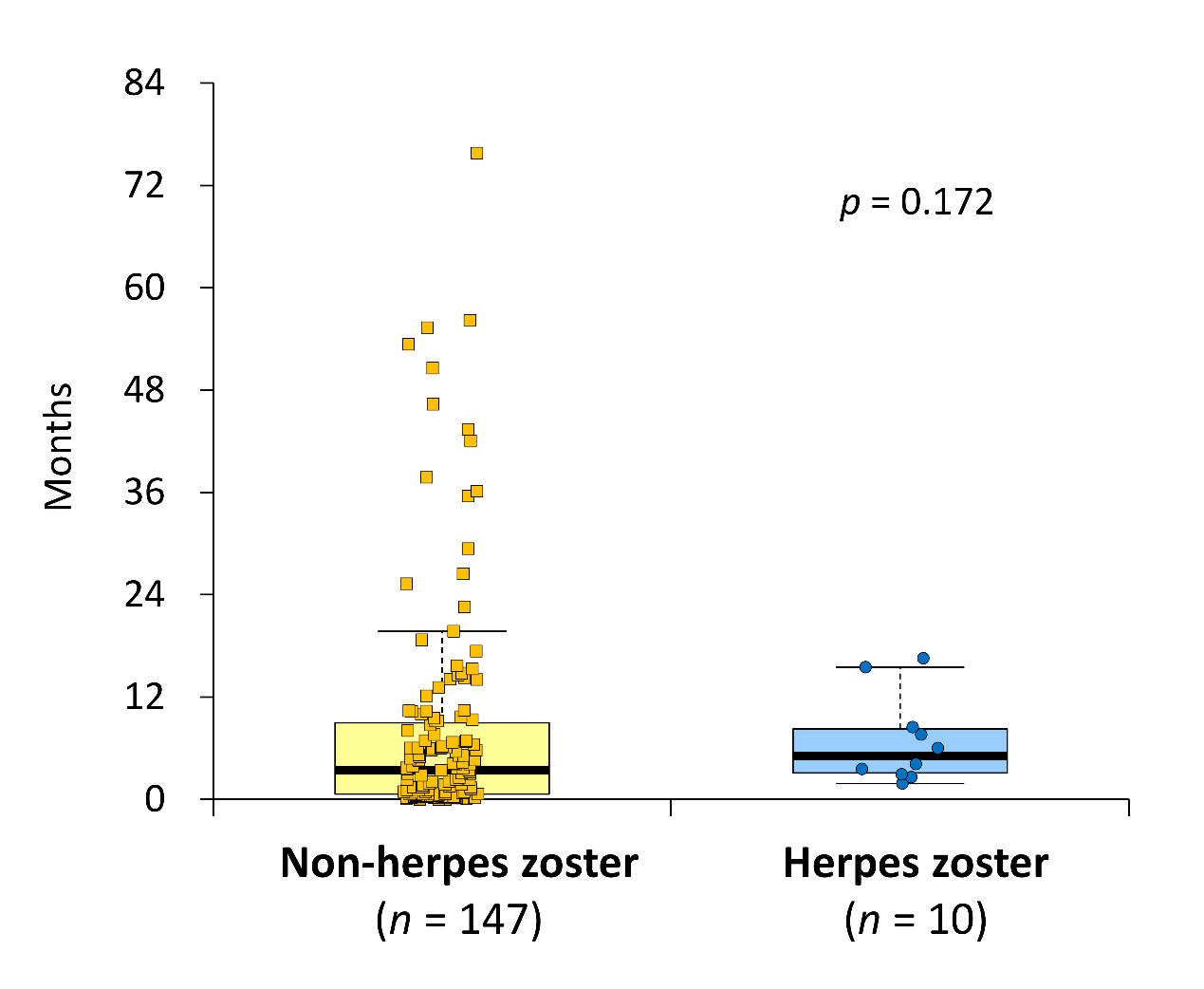

Supplement: Supplementary file 1 — Figure S1 [file CAM4-12-3013-s001.tif]
